# A Five-Year Survey for Plastic Surgery Malpractice Claims in Rome, Italy

**DOI:** 10.3390/medicina57060571

**Published:** 2021-06-03

**Authors:** Alessandro Feola, Chiara Minotti, Daniela Marchetti, Marco Caricato, Gabriella Teresa Capolupo, Luigi Tonino Marsella, Giuseppe La Monaca

**Affiliations:** 1 Department of Experimental Medicine, University of Campania “Luigi Vanvitelli”, Via Luciano Armanni 5, 80138 Napoli, Italy; 2 Bioethics and Humanities Research Unit, Campus Bio-Medico University of Rome, Via Alvaro del Portillo 21, 00128 Roma, Italy; chiaraminotti95@libero.it (C.M.); g.lamonaca@unicampus.it (G.L.M.); 3 Safety and Bioethics Department, Section of Legal Medicine, Catholic University of the Sacred Heart, Fondazione Policlinico “A. Gemelli” IRCCS, Largo Francesco Vito 1, 00168 Roma, Italy; daniela.marchetti@unicatt.it; 4 Colorectal Surgery Unit, Campus Bio-Medico University of Rome, Via Alvaro del Portillo 21, 00128 Roma, Italy; m.caricato@unicampus.it (M.C.); g.capolupo@unicampus.it (G.T.C.); 5 Department of Biomedicine and Prevention, “Tor Vergata” University of Rome, Via Montpellier 1, 00155 Roma, Italy; marsella.luigi@gmail.com

**Keywords:** medical liability, plastic surgery, medical jurisprudence

## Abstract

(1) Introduction: Medical malpractice claims against both health institutions and physicians are a crucial topic in Italy, as well as in other countries, particularly regarding civil proceedings. Our study reports an analysis of all of the malpractice judgments concerning plastic surgery decided in the Civil Court of Rome between 2012 and 2016. (2) Methods: the database of the Observatory Project on Medical Responsibility (ORMe) was analyzed, which collects all of the judgments of the Civil Court of Rome, that is, the first instance district court. Therefore, neither the jurisprudence of the second level court nor that of the Supreme Court was taken into account. (3) Results: 144 judgments concerning plastic surgery were delivered in the five-year period of 2012–2016 (corresponding to 10.6% of total professional liability verdicts of the Civil Court of Rome in the same period). In 101/144 cases (70.14%), the claim was accepted. A total of €4,727,579.00 was paid in compensation for plastic surgery malpractice claims, with a range from a minimum amount of €1555.96 to a maximum amount of €1,425,155.00 and an average compensation of €46,807.71 per claim that was significantly lower compared to other surgical disciplines. (4) Conclusions: Our data confirm that the analyzed branch has a high litigation rate, with a prevalence of convictions for cosmetic procedures over reconstructive ones, both for malpractice and for violation of the informed consent. Plastic surgery is also confirmed among those branches in which the professionals are more frequently sued compared to health institutions.

## 1. Introduction

Medical malpractice claims against both health institutions and physicians are a crucial topic in Italy, as well as in other countries, particularly regarding civil proceedings. With this regard, the European Academy of Legal Medicine (EALM) in 2013 proposed the European Guidelines on Medico-Legal Methods of Ascertainment and Evaluation Criteria in cases of suspected subjective medical responsibility and/or liability [1]. In Italy, the Law n. 24/2017, also named the Gelli-Bianco law [2,3], aimed to solve some critical issues of civil and criminal medical liability in order to produce a definitive clarification of the main aspects of healthcare professional liability, a better determination of the obligation to repair or satisfy the consequences of medical action from a criminal, civil, or administrative perspective, and a deflation of litigations.

Several years are needed to have evidence about the effects of this law from its entry into force; it can nevertheless be useful for an analysis on medical malpractice claims to highlight the effective pressure on sanitary systems, as well as to have an overview of the costs of health malpractice compensation.

Some Italian studies have examined the litigation trends for specific health branches in the district of Rome. Tarantino et al. [4] reported about malpractice judgments concerning orthopedics decided in the Civil Court of Rome between 2004 and 2010, analyzing 243 verdicts, of which 182 (75%) were found in favor of the plaintiff with a total indemnity payment of more than €12,350.00.

While Manca et al. [5] focused on malpractice claims in dentistry between 2001 and 2015 in the Civil Court of Rome, analyzing 458 verdicts among which, in 339 cases (74%), the dentist was judged as guilty with an average compensation granted of €18,820.15. Other studies were based on insurance experience in different geographical areas [6,7].

Our paper reports an analysis of all of the malpractice judgments concerning plastic surgery decided in the Civil Court of Rome between 2012 and 2016. We focused on plastic surgery, considering the high rate of malpractice claims in this area and its growth with the increase of available procedures [8,9,10,11]. We chose to carry out an analysis on jurisprudence, rather than on the data provided by insurance brokers. The limit of the former is not to intercept the compensation claims processed out of court, while offering the possibility of an effective monitoring of decisions of the Civil Court of Rome, which, according to the data of the Italian Ministry of Justice, represented in 2012 13.41% of overall new national civil proceedings [12]. Furthermore, this approach is less influenced by the commercial dynamics of the insurance market.

## 2. Materials and Methods

**Figure 1:**
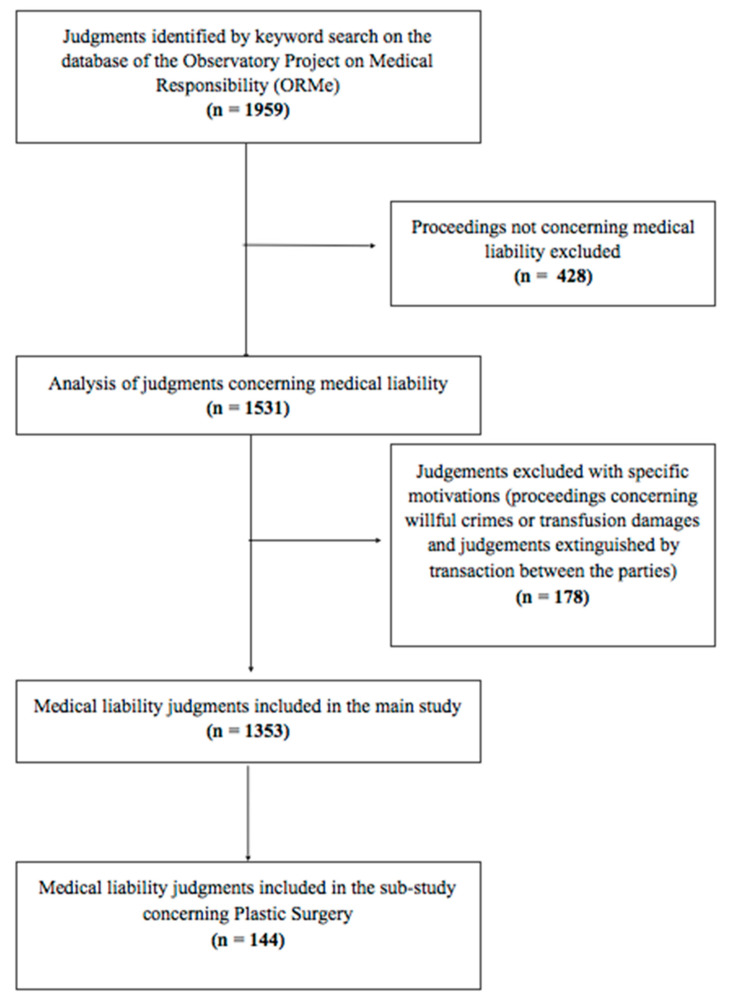
Flowchart about proceedings selection.

We analyzed the database of the Observatory Project on Medical Responsibility (ORMe), which collects all of the judgments of the Civil Court of Rome, that is, the first instance district court. Therefore, neither the jurisprudence of the second level court nor that of the Supreme Court was taken into account. A five-year period preceding the aforementioned Law n. 24/2017 (from 1 January 2012 to 31 December 2016) was selected, because we believe that a survey of the judgements about malpractice claims related to a period before its coming in turn could support any further speculation regarding the outcomes of the new law. Within the database, we researched all of the judgments concerning proceedings for medical malpractice liability, using the following keywords: “medico” or “medici” or “chirurgo” or “chirurghi” or “sanitari” or “ospedale” or “ospedali” or “policlinico” or “casa di cura” (doctor, doctors, surgeon, surgeons, healthcare professionals, hospital, hospitals, polyclinic, and nursing home). The judgments collected underwent a first screening excluding those not relevant to the issue of medical liability (Figure 1). Then, proceedings concerning willful crimes, transfusion damages, or damages from blood products, as well as judgments extinguished by transactions between the parties, were excluded; 1353 judgments were therefore included in the study. They were further classified by the health area involved, typology of defendants (physicians/health institution), type of damage claimed (injury/death), verdict, and compensation paid. Finally, 144 judgments concerning the area of plastic surgery were selected; this area was defined in agreement with the Italian Ministry of Health Decree of 4 October 2000, also including minor procedures sometimes classified as aesthetic medicine procedures. The 144 judgments were then analyzed by these further parameters: year of decision, type of treatment carried out, and relevance of informed consent in court decisions.

## 3. Results

One hundred and forty-four judgments concerning plastic surgery were delivered in the five-year period of 2012–2016 (corresponding to 10.6% of total professional liability verdicts of the Civil Court of Rome in the same period). Only the fields of orthopedics, general surgery, and gynecology and obstetrics had a higher number of proceedings in that period (respectively, 217, 177, and 168).

In 3/144 cases, plastic surgery was involved together with other medical branches: once with general surgery (a bilateral mastectomy with subsequent reconstructive mastoplasty, following a misdiagnosis of neoplasia), once with radiology, and once again with radiology, anesthesia, and intensive care (both cases concerning breast augmentation procedures without diagnosing breast cancer).

141/144 verdicts (97.92%) concerned claims for non-fatal injuries. Only 3/144 verdicts (2.07%) concerned fatal injuries (one verdict delivered in 2012, two in 2015): in one case, a transverse rectus abdominis myocutaneous (TRAM) flap procedure for breast reconstruction after mastectomy for cancer was performed in a 61-year-old patient, and it was complicated by severe anemia, ischemia of the flap, sepsis, myocardial infarction, and death approximately 50 days after the surgery; the second case concerned an abdominoplasty procedure, complicated by bleeding due to iatrogenic vascular lesion, that was ineffectively treated. In the third case, a patient had undergone the removal of a congenital flat hemangioma of the face, resulting in a deformation requiring subsequent operations and a severe depressive reaction, culminating in suicide 10 years after the first treatment.

In 101/144 cases (70.14%), the claim was accepted (Table 1); this indicates a higher percentage compared to the overall number of convictions recorded in the total five-year judgments for medical liability (58.5%). Only orthopedics exceeded the area of plastic surgery in the absolute number of convictions (135 claims accepted, but with a lower acceptance rate of 62.2%). In terms of the prevalence of convictions, plastic surgery clearly exceeded other surgical branches, with a high incidence of malpractice claims, like orthopedics, gynecology and obstetrics, and general surgery (respectively, 62.2%, 55.4%, and 51.4%).

The claim was accepted in all three fatal cases, as well as in one out of three where plastic surgery was involved together with other medical branches (radiology).

The following Table 2 and Table 3 show the distribution of citations and convictions between institutions and medical professionals.

In 84/144 proceedings (58.33%), both professionals and health institutions were sued, in 13/144 (9.03%) only health institutions, and in 47/144 (32.64%) only professionals. Health institutions were found liable in 57/144 verdicts (39.57%), specifically in 57/97 cases (58.75%) in which they were sued, with little prevalence of convictions for public institutions compared to private (64% vs. 53.1%). Professionals were found liable in 92/144 verdicts (63.89%), corresponding to 70.23% of 131 proceedings in which they were sued. Particularly, they were condemned in 37/47 proceedings (78.71%), in which they had been sued exclusively; in 40 of them, a single professional was sued (with 31 convictions), in seven judgments, more professionals had been sued (in one case, the claim was decided in favor of the defendants, in one other, only a professional was condemned, and in the remaining five cases, two or more professionals were found liable). Institutions were found liable in 8/13 proceedings (61.54%) in which they had been sued exclusively; in seven of those 13 proceedings, a single public health institution had been sued (with three verdicts favorable to the claimants). In the remaining six cases, a single private health institution had been sued (with five verdicts favorable to the claimants). Finally, in three proceedings, the manufacturer of the devices used was also sued, but was never condemned. A total of €4,727,579.00 was paid in compensation for plastic surgery malpractice claims between 2012 and 2016 (Table 4), with an average compensation of €46,807.71 per claim (considering only proceedings with a verdict favorable to the claimants) that was significantly lower compared to other surgical disciplines (general surgery = €276,577.21 per claim; gynecology and obstetrics = €290,591.64 per claim; orthopedics = €76,804.82 per claim). A total amount of €2,716,068.86 (with an average of €27,714.99) was paid for the proceedings with non-fatal injuries, while a total amount of €2,011,510.14 (with an average of €670,503.38) was paid for the three fatal claims.

In 49/101 cases (48.50%), a lack or invalidity of informed consent for treatment was relevant in the verdict of conviction.

The procedures performed in the 144 judgements have been classified according to the American Society of Plastic Surgeons criteria [13,14]. A total of 168 procedures were considered (some claims concerned multiple treatments applied to a single subject): 142 of cosmetic surgery and 26 of reconstructive surgery.

Table 5 and Table 6 show the cosmetic and reconstructive surgery procedures with the number of convictions.

## 4. Discussion

Several international studies analyzed malpractice in plastic surgery, focusing on judicial decisions in legal disputes. They assume that such investigation and a better knowledge of the motivations and mechanisms of the claims could allow a reduction of litigations and of the subsequent costs for health systems [8,10,15].

According to this aim, we carried out a descriptive study on Italian jurisprudence in this field, collecting all plastic surgery malpractice claims sentenced in a five-year period (2012–2016) by the Civil Court of Rome, in the first instance trial, as a representative sample. We analyzed the verdicts of a first instance court, because they are more numerous and significant in having a correct perception about litigation in plastic surgery, considering that, in the Italian legal system, higher courts examine only a minor number of initial disputes [16].

Our study has some limitations due to the analysis only of the text of the judgments and the impossibility to access other documents related to the proceedings and, particularly, the conclusions of the expert report, which is usually of crucial relevance in medical malpractice disputes. Furthermore, we were unable to determine the total number of plastic surgery and aesthetic medicine procedures performed during the study period; in fact, while the public hospital data are known, the procedures performed in private hospitals and in outpatient private facilities are not available. Therefore, we could not estimate the effective rate of procedures that led to claims. Finally, our data are restricted to Civil Court judgments, and we have no information regarding litigations settled outside of court. Nevertheless, our study provides a representative sample of civil litigation in Italy concerning plastic surgery, allowing us to deduce clear trends and extrapolate general considerations on the topic.

As expected, we reported a high prevalence of non-fatal injuries, and lethal outcomes were observed only in three cases, with a significantly lower prevalence than in global five-year malpractice litigation period (2.1% versus 14.6%).

The defendants were found liable in 70.14% of cases, more frequently than in other specialties with a high rate of litigation (only orthopedics had a higher absolute number of convictions). The percentage of convictions we found was higher than in global malpractice litigation in the same period (58.5%), even if compared to other international surveys concerning plastic surgery [9,10]. A recent study published in the United States by Therattil et al. [10] showed, in particular, that most of proceedings in plastic surgery (65.5%) resulted in favor of the defendant, and this more often happened in procedures like liposuction and body contouring, whereas breast surgery procedures were more likely to result in favor of the plaintiff. Similarly, Svider et al. [11], in a study limited to facial surgery, found a prevalence of verdicts in favor of the defendant (62.5%).

In our series, the most commonly litigated procedures involved breast surgery both with aesthetic and reconstructive purposes (41.1%), followed by face and neck surgery mainly represented by rhino/septoplasty (10.1% of all procedures). A prevalence of breast surgery procedures in litigations has also been reported by Therattil et al. (34.4%) [10]. A Brazilian court experience on medical disputes in plastic surgery [17] reported that most lawsuits pertained to breast surgery (32%), followed by abdominoplasty (24%), rhinoplasty (22%), and liposuction (22%) as other procedures frequently involved, and most complaints were about scarring or remodeling achieved (48.9%), general dissatisfaction with the outcome of the procedures (25.6%), or post-operative complications (25.5%).

In our series, claims were accepted more frequently for cosmetic than for reconstructive procedures (76.75% vs. 50%), and cosmetic procedures with a higher rate of verdicts favorable to the claimants were face and neck and fat reduction procedures.

Our study paid particular attention to the different kinds of defendants. In plastic surgery, most claims were against professionals in comparison with institutions (90.96% vs. 67.35%), different from what was observed in the overall medical litigation (including all areas) in the same period (64.4% vs. 85.4%). An exclusive involvement of health institutions was found in only 9.03% of cases (vs. 35.6% in the overall five-year malpractice litigation period), while professionals alone were sued more frequently than observed in the global five-year malpractice litigation period (32.64% vs. 14.6%). Private institutions were sued more often than public ones. A single professional was sued in 72.92% of claims, while more professionals were involved in only 18.05% of cases.

Convictions were prevalent among professionals rather than institutions both numerically (96 vs. 57) and in percentage rate (70.23% vs. 58.75%). Private health institutions were condemned more frequently than public ones. The three judgments concerning fatal injuries all resulted in a conviction (in one case, both the plastic surgeon and the health institution were found liable, in the other two, only the health institutions).

It was not possible to carry out an analysis of the compensations requested by the claimants, as this data was not reported in most verdicts. The average compensation paid for judgments concerning plastic surgery (€46,807.71) was significantly lower compared to that recorded for the global five-year malpractice litigation period (€191,177.15) with a maximum amount of €1,425,155.00. Such amounts are largely within the average ceilings offered by insurance policies in Italy for compensation for medical malpractice.

The invalidity or inadequacy of informed consent was a recurring subject of dispute, and was frequently included among the motivations of convictions (in 34% of judgments and 48.5% of convictions), especially for cosmetic procedures, confirming the importance of acquiring an adequate consent to the treatment and the need of effective preoperative communication with the patient in order to prevent litigation, as highlighted in other studies [17,18,19,20,21,22]. Particularly, Bismark et al. reported that, in 70% of cases, the complaints concerned insufficient information about the adverse effects and complications of procedures, in 39% of cases, the lack of information about the possibility of no benefit from treatment, and in 26% the inappropriate process of acquiring consent [21]. Da Silva et al. [22] highlighted that informed consent played a pivotal role in the legal outcome, as well as the quality of medical files and expert witness.

## 5. Conclusions

Our data confirm that plastic surgery has a high litigation rate, with a prevalence of convictions for cosmetic procedures over reconstructive ones, both for malpractice and for violation of informed consent. The need of a more balanced patient and physician relationship, as well as the attitude of some patients to engage in financial speculations after major or minor perioperative complications, can explain such data. Furthermore, the importance of effective and exhaustive communication with the patient must be stressed, especially in cosmetic procedures, correctly prospecting the limits and potential benefits of the treatment proposed without feeding improper expectations to the patient and documenting the information provided.

Plastic surgery is also confirmed among those branches in which the professionals are more frequently sued compared to health institutions. This trend will reliably not change significantly in the coming years under the effect of Law n. 24/2017, considering that plastic surgery is a medical specialty mainly practiced in a private setting, and therefore slightly affected by legislative changes introduced by the aforementioned law regarding the professional liability of physicians employed by public health institutions. The high conviction rate suggests the importance of strengthening all procedures for an amicable and out-of-court settlement of disputes.

## Figures and Tables

**Table 1:** Plastic surgery verdicts and convictions grouped by year.

	Verdicts	Convictions	%
2012	33	22	66.67%
2013	23	16	69.56%
2014	25	14	56.00%
2015	31	25	80.65%
2016	32	24	75.00%
Total	144	101	70.14%

**Table 2:** The distribution of the verdicts (and related convictions) with respect to health institutions. Institutions sued (private medical practices included) and related convictions. * In all six cases, a public institution was condemned; only in two of them was a private institution condemned too. ** The value does not include the seven proceedings in which both the professional and institution were cited with conviction only of the first.

	Verdicts	Convictions
Single public health institution	16 (11.10%)	10 (62.50%)
Single private health institution	65 (45.14%)	36 (55.37%)
Two or more public health institutions	0 (0.00%)	0 (0.00%)
Two or more health institutions (public and private)	9 (6.25%)	6 * (66.67%)
Two or more private health institutions	7 (4.85%)	5 (71.43%)
Total institutions sued	97 (67.35%)	57 (58.75%)
Institutions not sued	47 (32.64%)	37 (78.71%)
Total verdicts	144	94 **

**Table 3:** Professionals sued and related convictions. * The value does not include one proceeding in which both the professional and institution were cited with conviction only of the second.

	Verdicts	Convictions
Single professional	105 (72.92%)	74 (70.48%)
Two or more professionals	26 (18.05%)	18 (69.22%)
Total professionals sued	131 (90.96%)	92 (70.23%)
Professionals not sued	13 (9.03%)	8 (61.54%)
Total verdicts	144	100 *

**Table 4:** The distribution by year of the compensations in euros for the area of plastic surgery.

	Total	Mean	Minimum	Maximum	Total Amount for Non-Fatal Claims	Average Amount for All Areas
2012	874,303.43	39,741.07	1555.96	383,900.00	490,403.43	209,223.36
2013	432,016.27	27,001.02	2962.93	156,203.89	432,016.27	209,630.60
2014	348,448.82	24,889.20	2079.89	49,798.33	348,448.82	173,726.23
2015	2,290,715.81	91,628.63	1891.08	1,425,155.00	663,105.67	177,609.96
2016	782,094.67	32,587.28	3597.36	192,415.60	782,094.67	177,660.61

**Table 5:** Cosmetic surgery procedures.

Category of Treatment	Procedure	Verdicts (Convictions)
Breast	Augmentation	35 (22)
	Implant revision	5 (5)
	Lift	11 (8)
	Reduction	10 (9)
	Total	61 (44)
Fat reduction	Liposuction	11 (9)
	Nonsurgical fat reduction	1 (1)
	Total	12 (10)
Body Lifts	Arm lift	1 (1)
	Buttock Enhancement	2 (0)
	Thigh Lift	2 (2)
	Tummy Tuck	10 (7)
	Total	15 (10)
Face & Neck	Brow Lift	3 (3)
	Buccal fat removal	1 (1)
	Chin surgery	2 (1)
	Otoplasty	2 (1)
	Eyelid Surgery	5 (5)
	Facelift Surgery	2 (1)
	Facial Implants	3 (3)
	Neck Lift	4 (4)
	Rhinoplasty	9 (8)
	Total	31 (27)
Minimally invasive	Botulinum Toxin	1 (1)
	Dermal Fillers	14 (10)
	Laser Hair Removal	2 (2)
	Laser Skin Resurfacing	1 (1)
	Skin Rejuvenation and Resurfacing	1 (1)
	Tattoo Removal	1 (1)
	Spider Vein Treatment	1 (1)
	Total	21 (17)
Male-specific plastic surgery	Hair Transplant	1 (1)
	Men and Plastic Surgery	1 (0)
	Total	2 (1)
Overall Total		142 (109)

**Table 6:** Reconstructive surgery procedures.

Procedure	Verdicts (Convictions)
Breast Reconstruction	7 (4)
Breast Reduction	1 (0)
Congenital Anomalies	2 (2)
Gender Confirmation Surgeries	1 (0)
Hand Surgery	1 (0)
Septoplasty	8 (5)
Skin Cancer Removal	6 (2)
Total	26 (13)

## Data Availability

The data presented in this study are available on request from the corresponding author. The data are not publicly available due to privacy restriction.
